# Prevalence and correlates of early sexual initiation among Brazilian adolescents

**DOI:** 10.1371/journal.pone.0260815

**Published:** 2021-12-14

**Authors:** Alejandra Andrea Roman Lay, Elizabeth Fujimori, Luciane Simões Duarte, Ana Luiza Vilela Borges

**Affiliations:** 1 Faculty of Health Sciences, University of Tarapacá, Arica, Chile; 2 Public Health Nursing Department, School of Nursing, University of São Paulo, São Paulo, Brazil; Public Library of Science, UNITED KINGDOM

## Abstract

**Background:**

Early adolescence is a critical stage in adolescents. This is the phase at which many young people start their sexual life early, increasing the risk of sexually transmitted infections and unintended pregnancy. Few studies have analyzed the factors associated with early sexual initiation in very young adolescents in low- and middle-income countries. Therefore, this study assessed the early sexual initiation stratified by sex and its correlates in a sample of Brazilian adolescents.

**Methods:**

The study sample included Brazilian adolescents aged 12–14 who participated in The Study of Cardiovascular Risk in Adolescents (ERICA), a multicenter, school-based, country-wide, cross-sectional study. Early sexual initiation was defined as the first sexual intercourse at or before 14 years old. Multivariate logistic regression was used to identify independent factors associated with early sexual initiation.

**Results:**

The prevalence of early sexual initiation was 7% among girls and 18% in boys. In a multivariate analysis, the adolescent’s age, mother’s schooling, smoking, alcohol consumption, and Tanner Stages were associated with early sexual initiation for both boys and girls. For girls, living with both parents, common mental disorders, and age at menarche were predictors of early sexual initiation, while race and type of school were correlated to early sexual initiation only for boys.

**Conclusions:**

The early sexual initiation was associated with sociodemographic, lifestyle risk factors and secondary sexual characteristics in both sexes, while there were differences between the predictors of early sexual initiation among girls and boys. It emphasizes the importance of sex education and promotes healthy lifestyles in environments through families and schools in early adolescence.

## Introduction

The early adolescence, between the ages of 10 and 14, also known as very young adolescents (VYA), is marked by significant physical growth, and it is when the sexual experimentation often begins. During these years, early sexual initiation (ESI), defined as the first sexual intercourse at or before the age of 14, which can be seen as a critical milestone for many adolescents on the trajectory to adulthood, can occur [1]. The prevalence of ESI varies according to sex, and, irrespective of the context, male adolescents are more likely to report ESI than females. During 2015 in the United States (U.S.), the early sexual debut was 27.3% among boys and 20.7% among girls [2], while, in Brazil, the prevalence of ESI is higher than the average in Latin America (18.6% versus 11%) but the difference between boys and girls remain [3, 4]. The high variability of ESI across countries can be explained by the scope of factors that influence an adolescent to have the first sexual intercourse. Some of the factors that are associated with an increased risk of ESI are black race/ethnicity [2], adoption of risky behaviors (e.g., tobacco and alcohol consumption) [5], being victims of violence and negligence [6] and having mothers with low educational attainment [7]. Furthermore, in adolescence, emotional maturity is incomplete and adolescents become vulnerable to the influence of their peers, including sexual initiation. Thus, being bulled in girls and internalized problems (i.e., emotional difficulties and peer problems) in boys are related to ESI [8].

ESI has been associated with risky behaviors such as inconsistent use or no use of contraceptives in the first or other sexual intercourses [9], multiple sexual partners and drug-use in the last sexual relation [10]. Therefore, ESI has negative impacts on adolescent sexual and reproductive health (SRH), increasing the risk of sexually transmitted infections (STIs) [11] and teenage pregnancy [12], especially for the VYA, since they are more exposed to the consequences of nonconsensual intercourse [13].

Previous Brazilian studies have been carried out to analyze sexual initiation in adolescents, however none of them have determined the factors associated with early sexual initiation. A cohort study conducted in Brazil during 2008 showed significant differences between early sexual initiation according to prevalence of sociodemographic factors and risk behaviors between boys and girls; however, the directions of the associations were not analyzed [3]. Another study with Brazilian adolescents with mean age of 14 years used Cox regression models to identify factors associated with sexual initiation, including age of sexual initiation as continuous variable, therefore it is not possible to analyze specifically those who initiated sex activity earlier than 15 years [14].

More than half of VYA are living in countries with poor SRH education and have pronounced gender differences in their socialization [15]. In the face of the possible adverse health outcomes of ESI to health, especially in developing countries, we assessed the prevalence and correlates to early sexual initiation at or before 14 years stratified by sex among adolescent aged between 12 to 14 years residents in some of the largest cities in Latin America.

## Methods

### Study design and sample

We use data from the Study of Cardiovascular Risks in Adolescents (Portuguese acronym, ERICA), conducted in Brazil in 2014. ERICA is a multicenter, school-based, countrywide, cross-sectional study representative of Brazilian adolescents. ERICA aimed to determine the prevalence of metabolic syndrome and obesity and to analyze cardiovascular risk factors among adolescents living in cities with more than 100,000 inhabitants.

ERICA is a complex sample. Firstly, a geographical stratification was carried out, considering 32 strata: 27 Brazilian capital states and the Federal District were selected to be representative of large cities at the national level, and five macroregions of the country were selected to be representative at the regional level. Secondly, 1,247 schools were selected based on the number of students in eligible classes and on the distance from the state capital. Schools were included according to situation (urban or rural areas) and governance (private or public). Using class year as a proxy of age, only grades seven (adolescents aged 12 years), eight (adolescents aged 13 years) and nine (adolescents aged 14 years) of elementary school, and the first (adolescents aged 15 years), second (adolescents aged 16 years) and third (adolescents aged 17 years) years of high school were eligible for selection. Third, the selection of school classes was conducted using different combinations of scheduled time at school (morning and afternoon). Sample design and protocol details are described elsewhere [16]. The inclusion criteria were adolescents aged 12 to 17 years. The exclusion criteria were pregnant girls, or individuals with any temporary or definitive disability that would make the adolescent unable to participate in the study (visual impairment, hearing impairment, physical disability or apparent intellectual disability) [16].

To calculate sample size, a prevalence of the metabolic syndrome in adolescents was 4%, considering a maximum absolute error of 1% and a confidence level of 95%. This represents a simple random sample size of 1,475 adolescents. However, ERICA’s sample is clustered by school and class. Thus, a design effect of three was used, which showed a sample size of 4,425 adolescents. As the estimates with controlled precision had to be produced for 12 domains (six ages X two sexes), it was estimated that the overall sample size would have 74,340 adolescents [16].

Finally, ERICA collected data of 74,589 adolescents. However, we excluded 1,254 adolescents who responded that they did not know or remember their age of sexual initiation, 179 adolescents who incorrectly answered the age of their first sexual relationship (e.g., an adolescent who was 14 years old and answered that their age of sexual debut was 17 years old), and 39,623 adolescents who were between 15–17 years. Therefore, the sample of the current study included 33,533 adolescents (14,960 males and 18,573 females).

### Ethics approval and consent to participate

ERICA was approved by the Committee for Ethics in Research (CEP) of the Institute of Collective Health Studies, Universidade Federal do Rio de Janeiro (Process 45/2008) and by a CEP of each of the 27 units of the Federation, states and Federal District. All adolescents who participated in the study signed an informed assent and had to bring an informed consent term signed by parents/guardians when required by the local Ethics Committees.

### Data collection

Trained evaluators invited the students from the selected classes to participate in the research and also explained the study and the questionnaire. The ERICA questionnaire was self-applied through a Personal Digital Assistant (PDA), model LG GM750Q. That privacy provided to answer the questions was important to guarantee the truth of the answers. Besides, that system allowed for no omission in the answers. Due to the sensitive nature of some questions, the answer options "I don’t know", "I don’t remember" and "I don’t want to answer" were included as unknown data.

### Measures

Our outcome was ESI, and the measure was assessed with the question, "How old were you when you had your first sexual intercourse?”. The sexual initiation was coded as 1 (early) if adolescents reported the age of debut before or at 14 years and 0 if sex has not been initiated. The independent variables were adolescent’s sociodemographic, health-related, and reproductive characteristics. The sociodemographic variables were age in years (12, 13, 14), race (white, black, Asian/indigenous/unknown), type of school (public, private), family structure (live with father or other relatives, only with mother, with both), mother’s education (elementary school, high school, university, unknown–these categories included complete and incomplete education) and wealth index (in quintiles) [17].

Health-related variables were the frequency of smoking and alcohol consumption, and mental disorders. Smoking was assessed with the question “In the last 30 days, in the days that you smoked, how many cigarettes did you smoke on average?”, and the adolescents were categorized according to the WHO indicator of current smoking cigarettes, i.e., smoking cigarettes at least once during an established period. Thus, the categories were never smoked/do not smoke, smoked (≤1 to ≥30 cigarette per day) or unknown (cannot remember how many cigarettes per day). The alcohol consumption was based on the question “In the last 30 days, on the days that you had an alcoholic drink, how many glasses or doses did you drink on average?”, and the adolescents were categorized according to the WHO indicator of alcohol consumers, 13–15 years old, i.e., consumption of alcohol at least once in the past 30 days. Thus, the categories were: never tried/do not drink, drank (≤1 to ≥5 doses) or unknown (cannot remember how many glasses or doses). Regarding mental disorders, the common mental disorders (CMD) were assessed through the 12-item General Health Questionnaire (GHQ-12). The GHQ-12 questions are related to the ability to concentrate, sleeping problems, decision-making problems, perceived stress, self-confidence, depression or perceived happiness. Each question has four answers: not at all, no more than usual, a little more than usual and much more than usual. The scores of these 12 items were coded individually as “absent” (not at all and no more than usual) or “present” (a little more than usual and much more than usual) dichotomized in 0 or 1 respectively and then summed up. Adolescents with a score of three or more were classified as cases of CMD. In a previous Brazilian study, where the GHQ-12 was validated, the criterion of 3 points or more for CMD was used, showing a sensitivity of 85%, specificity of 79% and area under the ROC curve of 0.87 [18].

The reproductive variables were Tanner Stages and the age of menarche. Tanner stages were self-assessed by images where the adolescents had to identify what stage (1 to 5) they were in according to the characteristics of breast development for girls and genital development for boys. We used tanner stage 4 in both sexes as the reference group for the statistical analysis. The age of menarche was self-reported and categorized as “does not have”, <12 years or ≥12 years.

### Data analysis

The complex sample weights of the ERICA survey design were accounted for all statistical analyses. The description of variables was presented with weighted-percentages and the differences between baseline variables by sex were evaluated using the Rao-Scott chi-square test. We used bivariate and multivariable logistic regression models to calculate Odds Ratios (OR) and 95% Confidence Intervals (CI) for the associations of socio-demographic, health-related, and reproductive variables to early sexual initiation, stratified by sex. In the final multivariate models, all study variables based on epidemiological plausibility and statistical significance were included. All analyses were performed using STATA 14, and p-values of <0.05 were considered statistically significant.

## Results

### Baseline characteristics stratified by sex

The median age of adolescents was 13.15 (SD 0.78). Of all adolescents, 7% of girls and 18% of boys reported ESI. The majority of adolescents were black (55%), were attending public schools (80%), lived with both parents (59%), had no mental disorders (73%), reported that never smoked/do not smoke (97%), neither used alcohol (79%), and their mothers reported not knowing their educational level (34%). The highest proportion of adolescent girls was found in quintile 1 of wealth index (24%), while in boys it was found in quintile 5 (24%). Regarding reproductive variables, most adolescents were between Tanner Stage 3 and 4 (65% girls and 66% boys), and 41% of the girls presented early menarche (<12 years). There were sex differences according to race, family structure, mother’s education, wealth index, alcohol consumption, and common mental disorders (p <0.005) (**Table 1**).

**Table 1:** Baseline adolescent’s characteristics stratified by sex, ERICA study, Brazil (n = 33,533).

Baseline characteristics	All sample (n = 33,533)	Girls (n = 18,573)	Boys (n = 14,960)	
	%^a^	%^a^	%^a^	p-value^b^
Age				
12	33.38	33.37	33.38	0.99
13	33.23	33.19	33.26	
14	33.39	33.44	33.35	
Race				
White	39.08	38.22	39.93	<0.001
Black	55.12	57.40	52.87	
Asian/indigenous/unknown^c^	5.80	4.37	7.20	
Type of school				
Public	79.52	80.19	78.87	0.10
Private	20.48	19.81	21.13	
Family structure				
With father or other relatives	9.38	9.42	9.34	0.002
Mother	31.71	33.99	29.47	
With both parents	58.91	56.59	61.19	
Mother’s education^d^				
Elementary school	23.29	23.50	23.09	<0.001
High school	21.31	22.36	20.28	
University	21.52	19.58	23.44	
Unknown^c^	33.87	34.57	33.18	
Wealth Index (quintiles)				
Q1	21.40	24.09	18.76	<0.001
Q2	18.28	18.78	17.79	
Q3	17.58	17.75	17.41	
Q4	20.64	19.02	22.24	
Q5	22.10	20.36	23.80	
Frequency of smoking				
Never smoked/do not smoke	96.56	96.24	96.88	0.20
Smoke^e^	2.54	2.71	2.39	
Unknown^c^	0.89	1.05	0.73	
Frequency of alcohol consumption				
Never experienced/do not drink	78.65	77.04	80.23	0.002
Drink^f^	15.24	16.61	13.89	
Uknown^c^	6.11	6.35	5.88	
Common mental disorders				
None	73.36	65.57	81.00	<0.001
3 or more	26.64	34.43	19.00	
Tanner Stage^g^				
1		2.83	3.67	
2		14.02	15.76	
3		33.44	22.78	
4		31.85	43.69	
5		17.86	14.10	
Age at menarche*				
Does not have yet		15.28		
<12		42.33		
≥12		42.39		

^a^ Relative distribution according to complex sampling design of ERICA study.

^b^ Rao-Scott Chi-Square Test.

^c^ Don’t know/don’t want to answer.

^d^ Elementary school: without studies, one to seven years (incomplete) and complete; High school: incomplete and complete; University: incomplete and complete.

^e^ In the last 30 days, ≤1 to ≥30 cigarettes per day.

^f^ In the last 30 days, ≤1 to ≥5 doses.

^g^ Breast development stage for girls and genital development stage for boys.

* Variable exclusive of girls with the exclusion of unknown responses, total sample size n = 18.228.

### Correlates of ESI

In the multivariable logistic regression analysis, being older ≥13, mother’s education, smoking, alcohol consumption and Tanner Stages were associated with ESI in both sexes. Girls whose mothers had university education (OR = 0.62, 95% CI:0.41,0.95) and boys whose mothers had high school (OR = 0.75, 95% CI:0.58,0.97) and unknown education (OR = 0.78, 95% CI:0.63,0.97) were less likely to ESI, compared to adolescents whose mothers had elementary school. Girls who reported smoking had a higher chance of ESI compared to boys who reported smoking (OR = 6.14 95% CI = 3.93,9.58 vs. OR = 5.12 95% CI:3.07,8.53). While those girls with unknown smoking (OR = 5.09 95% CI = 2.10,12.29) had a similar chance of ESI than boys who smoked. Both girls and boys who reported drinking (OR = 3.53 95% CI = 2.72,4.56 vs. OR = 4.09 95% CI:3.32,5.05) and those who reported not knowing if they drank (OR = 1.65 95% CI = 1.14,2.39; OR = 2.05 95% CI:1.36,3.08, respectively), had a higher odds of ESI than adolescents who do not drink. Regarding Tanner Stages, girls and boys who presented Tanner Stage 5 had almost twice the odds of ESI (OR = 1.52 95%CI:1.13,2.04; OR = 1.56 95%CI:1.27,1.91, respectively), while boys who presented Tanner Stage 1 were less likely to ESI (OR = 0.49 95% CI:0.29,0.84) compared to adolescents with Tanner Stage 4.

In girls, living with both parents was associated with lower odds of ESI (OR = 0.56 95% CI:0.40,0.77), while those with 3 or more CMD had 1.91 times increased odds of ESI (OR = 1.91 95% CI = 1.47,2.49) compared to their references. Regarding the age of menarche, girls who had menarche before 12 years (OR = 7.39 95% CI:3.64,14.99) and those with age at menarche at 12 or more years (OR = 3.62 95% CI:1.77,7.39) had higher odds of ESI versus girls without menarche.

In boys, those who self-reported being black and Asian/indigenous had an increased odds of ESI by 38% and 59% compared to whites boys (OR = 1.38 95% CI: 1.12,1.70; OR = 1.59 95% CI: 1.19,2.13, respectively). On the contrary, boys who attended private schools were less likely to ESI (OR = 0.49 95% CI:0.34,0.70) compared to peers attending public schools (**Table 2**).

**Table 2:** Unadjusted and adjusted Odds Ratios (OR) and 95% confidence intervals (CI) of the associations between early sexual initiation (ESI) and adolescent’s characteristics in girls and boys, ERICA study, Brazil.

	Girls (n = 18,573)						Boys (n = 14,960)						
Variables	Unadjusted model			Adjusted model^a^			Unadjusted model			Adjusted model^a^			
	OR	95% CI	p-value	OR	95% CI	p-value	OR	95% CI	p-value		OR	95% CI	p-value
Age													
12	1.00	Reference		1.00	Reference		1.00	Reference			1.00	Reference	
13	5.25	3.75,7.36	<0.001	4.07	2.65,6.26	<0.001	1.91	1.51,2.42	<0.001		1.79	1.38,2.32	<0.001
14	15.03	11.04,20.46	<0.001	10.31	7.22,14.71	<0.001	3.22	2.47,4.20	<0.001		2.75	2.09,3.62	<0.001
Race													
White	1.00	Reference		1.00	Reference		1.00	Reference			1.00	Reference	
Black	1.20	0.98,1.48	0.074	1.06	0.78,1.43	0.707	1.49	1.25,1.78	<0.001		1.38	1.12,1.70	0.002
Asian/indigenous/unknown^b^	0.85	0.58,1.25	0.426	0.74	0.44,1.26	0.271	1.43	1.11,1.83	0.005		1.59	1.19,2.13	0.002
Type of school													
Public	1.00	Reference		1.00	Reference		1.00	Reference			1.00	Reference	
Private	0.37	0.23,0.59	<0.001	0.66	0.42,1.03	0.069	0.36	0.25,0.52	<0.001		0.49	0.34,0.70	<0.001
Family structure													
With father or other relatives	1.00	Reference		1.00	Reference		1.00	Reference			1.00	Reference	
Mother	0.89	0.62,1.28	0.547	0.87	0.58,1.31	0.502	0.94	0.67,1.30	0.720		1.12	0.69,1.82	0.646
With both parents	0.46	0.34,0.63	<0.001	0.56	0.40,0.77	<0.001	0.63	0.47,0.85	0.003		0.82	0.52,1.29	0.398
Mother’s education^c^													
Elementary school	1.00	Reference		1.00	Reference		1.00	Reference			1.00	Reference	
High school	0.87	0.64,1.19	0.398	0.97	0.69,1.38	0.882	0.67	0.53,0.84	0.001		0.75	0.58,0.97	0.029
University	0.44	0.28,0.67	<0.001	0.62	0.41,0.95	0.028	0.65	0.49,0.86	0.004		1.00	0.76,1.30	0.990
Unknown^b^	0.71	0.54,0.93	0.014	0.94	0.67,1.32	0.733	0.72	0.60,0.85	<0.001		0.78	0.63,0.97	0.032
Wealth index (quintiles)													
Q1	1.00	Reference		1.00	Reference		1.00	Reference			1.00	Reference	
Q2	0.82	0.60,1.12	0.223	1.05	0.74,1.50	0.773	1.33	0.98,1.79	0.065		1.33	0.99,1.79	0.062
Q3	0.58	0.44,0.79	0.001	0.80	0.58,1.11	0.183	1.03	0.78,1.37	0.805		1.24	0.92,1.67	0.163
Q4	0.48	0.34,0.67	<0.001	0.71	0.49,1.01	0.060	0.69	0.51,0.93	0.016		0.89	0.64,1.23	0.472
Q5	0.51	0.36,0.73	<0.001	0.80	0.56,1.13	0.206	1.01	0.76,1.34	0.929		1.26	0.89,1.79	0.187
Frequency of smoking													
Never experienced/do not smoke	1.00	Reference		1.00	Reference		1.00	Reference			1.00	Reference	
Smoke^d^	15.41	10.62,22,36	<0.001	6.14	3.93,9.58	<0.001	8.74	5.52,13.84	<0.001		5.12	3.07,8.53	<0.001
Unknown^b^	11.59	6.12,21.95	<0.001	5.09	2.10,12.29	<0.001	2.54	0.98,6.56	0.053		1.72	0.55,5.41	0.352
Frequency of alcohol consumption													
Never experienced/do not drink	1.00	Reference		1.00	Reference		1.00	Reference			1.00	Reference	
Drink^e^	6.53	5.2,8.15	<0.001	3.53	2.72,4.56	<0.001	5.46	4.34,6.88	<0.001		4.09	3.32,5.05	<0.001
Unknown^b^	3.03	2.18,4.22	<0.001	1.65	1.14,2.39	0.008	2.77	1.95,3.93	<0.001		2.05	1.36,3.08	0.001
Common mental disorders													
Does not have	1.00	Reference		1.00	Reference		1.00	Reference			1.00	Reference	
3 or more	2.72	2.18,3.41	<0.001	1.91	1.47,2.49	<0.001	1.49	1.19,1.87	<0.001		1.17	0.91,1.50	0.209
Tanner Stage^f^													
1	0.53	0.23,1.21	0.136	0.96	0.40,2.32	0.925	0.66	0.40,1.08	0.102		0.49	0.29,0.84	0.010
2	0.50	0.32,0.78	0.003	1.06	0.64,1.73	0.820	0.76	0.57,1.00	0.052		0.98	0.74,1.29	0.888
3	0.68	0.52,0.87	0.003	0.97	0.72,1.31	0.858	0.85	0.68,1.07	0.188		0.85	0.65,1.11	0.242
4	1.00	Reference		1.00	Reference		1.00	Reference			1.00	Reference	
5	2.05	1.59,2.64	<0.001	1.52	1.13,2.04	0.006	1.86	1.52,2.28	<0.001		1.56	1.27,1.91	<0.001
Age at menarche*													
Does not have yet	1.00	Reference		1.00	Reference								
<12	20.73	11.42,37.62	<0.001	7.39	3.64,14.99	<0.001							
≥12	14.28	7.65,26.65	<0.001	3.62	1.77,7.39	<0.001							

^a^ Adjusted by all the variables shown in table.

^b^ Don’t know/don’t want to answer.

^c^ Elementary school: without studies, one to seven years (complete) and complete; High school: incomplete and complete; University: incomplete and complete.

^d^ In the last 30 days, ≤1 to ≥30 cigarettes per day.

^e^ In the last 30 days, ≤1 to ≥5 doses.

^f^ Breast development stage for girls and genital development stage for boys.

* Variable exclusive of girls with the exclusion of unknown responses, total sample size n = 18.228.

## Discussion

Using a large sample of adolescents from Brazil, we found that adolescent’s age, mother’s education, frequency of smoking and alcohol consumption, and Tanner Stages were associated to ESI in boys and girls. Among girls, the predictors of ESI were living with both parents, having three or more common mental disorders, being on scale 5 of the breast development of the Tanner Stages, and having initiated menarche. For boys, to be black and Asian/indigenous/unknown race, to attend in public school and to be on scales 1 and 5 of the genital development of the Tanner Stages were predictors of ESI. To our understanding, this is the first study in Latin America analyzing factors associated to ESI among the VYA. Our results bring new contributions to the knowledge about the individual, reproductive and behavioral risks of ESI among the youngest in a country with high levels of gender, education, and economic inequality.

Our study observed that the prevalence of ESI varies by skin color, as the literature has already shown. For instance, in the United States, a study with two samples of adolescents carried out in the same city, but in different schools, showed a total prevalence of 36.5% of ESI, however, the percentage was higher among white adolescents (47.2%), followed by African American (25.6%), native (5.3%) and Asian (21.9%) [19]. Another recent study that assessed only adolescent boys found that non-Hispanic black and Hispanics had a higher proportion of sexual onset before 13 years old, compared to non-Hispanic white [7]. In the same way, our results showed that non-white boys had higher odds of ESI than white boys. In Brazil, these differences in ethnicity indicate the influences of different socioeconomic groups, sociodemographic contexts and regional values.

Attending private schools delayed early sexual initiation in boys. There are few studies differentiating ESI between public and private schools. In Brazil, the type of school reflects the socioeconomic family’s status, but our data does not specify the family income and, consequently, does not explain the socioeconomic effects on ESI. For this reason, we decided to include wealth index as a measure of socioeconomic status, however this indicator was not statistically significant. Our results reinforce the importance of sexual education at schools [20], especially in public schools.

Family is the first social bond of the adolescent, and this family environment provides adolescents with the initial knowledge and values about sex. Therefore, it is no surprising that diverse studies show that different parenting style (i.e., strategies used to raise the children, as authoritarianism and permissiveness), lack of communication with parents and family structure, are predictors of ESI [20]. Regarding the family structure, there are protective factors that help delay sexual debut in adolescents, such as living in a dual-parent family [21], as well as maintaining closer communication with parents (girls with their fathers and boys with their mothers) and, on the contrary, the absence of the father increases the risk of ESI in girls [22]. Furthermore, adolescents with a close relationship with mothers who disapprove ESI are less likely to initiate sexual activity [23].

In this home context, some studies suggest that maternal education delays sexual initiation in both sexes [24], similar to our results. This association has been found to be stronger in the mother-daughter relationship than in the mother-son relationship [25]. Mothers with high levels of education can provide their children with a better sexuality education and lead them to safer sex in the first sexual intercourse, preventing adverse consequences such as unintended pregnancy and sexually infectious diseases [20].

Regarding risk behaviors, it is widely known that the frequency of smoking and alcohol consumption has negative effects on sexual initiation in adolescents [5, 8]. Early tobacco use increases the risk of alcohol consumption and early sexual debut in adolescents younger than 15 years [26]. Thus, the use of these two substances–which are related to each other–could increase adolescent’s ESI. The mechanism by which alcohol consumption leads to ESI has been explained through adolescent behavioral and social disinhibition [19]. Additionally, having sex under the influence of alcohol increases the risk of unsafe sexual intercourse, STIs, and unintended pregnancy [27]. In Brazil, the rate of alcohol consumption among adolescents is high. The prevalence of drinking among adolescents aged 13 to 16 years was 25% in boys and 27% in girls [28]. Added to the high consumption of alcohol, smoking is also prevalent among Brazilian adolescents, reaching 8% in national surveys [29].

Among girls, the presence of common mental disorders increased the odds of ESI. Some studies have shown an increment of depression symptoms after early sexual onset in Brazilian adolescent girls [30]. However, few studies have analyzed the influence of mental disorders on ESI prior to sexual initiation. One study has found that adolescents with mental disorders such as depression and antisocial disorders were more likely to report ESI [31].

It is known that early puberty has been associated with ESI in both male and female adolescents. Furthermore, precocious puberty has been associated with an increase of violence for boys and other adverse health outcomes for girls, such as bullying [32], delinquency, depressive symptoms [33], early pregnancy [34] and alcohol consumption [32], and, consequently, unsafe sex and sexually transmitted infections [27]. Our findings showed that girls who started their menstruation were more likely to report ESI; those who experienced early menarche had twice the odds of ESI compared to girls with menarche at or over 12 years. In particular, girls may be at greater risk of negative sexual, reproductive and social consequences of early puberty increasing gender inequality at a young age.

### Limitations

This study has limitations. First, we did not have information about the partner with whom the adolescents engaged in had their ESI and under which circumstances. This information is relevant because, in Brazil, of the total cases of sexual violence that occurred between the years 2011–2017, 40.5% of the total sexual violence cases were committed against children and adolescents. Among adolescent cases, 68% were between 10–14 years old and 92% were girls. Thus, sexual initiation at a very young age could be involuntary, related to coercion or violence, against sexual and reproductive rights [35]. Secondly, religion has been associated with a delay in the initiation of sexual activity among Brazilian adolescents [36]. Therefore, there may be a residual confounding in our results due to the non-inclusion of religion that was not evaluated in adolescents. Third, the questionnaire was self-reported, and since the PDA did not admit any missing data, we found many “do not know” answers for some variables, such as alcohol consumption (6%) and mother’s education (27%). In that way, many sensitive questions may have been under or overestimated according to the individual’s perception or experience. Fourth, a cross-sectional study does not allow the full understanding of some associations, such as that between the alcohol use and ESI, as one may overlap or precede the other. Nevertheless, the literature shows that alcohol consumption can predict sexual initiation [27], as well as ESI, which in turn can lead to alcohol consumption in adolescents [37]. Finally, it is important to consider that the data of this study were collected in 2014. However, the sociodemographic, health and risk factors associated with early sexual initiation in Brazilian adolescents in this study could have worsened since then, due to the economic recession in Brazil during 2015/2016, alongside with a political crisis [38, 39] and then by the Covid-19 pandemic [40, 41], that includes schools dropout, which is usually refereed as a protection against sexual risky behaviors [42, 43].

## Conclusions

Adolescent’s age, mother’s education, frequency of smoking, alcohol consumption, Tanner Stages were correlated with ESI regardless if boy or girl. There were differences in factors associated with ESI according to sex, among girls living with both parents, having three or more CMD and onset of menarche, while among boys, differences were found regarding race and attending public schools. Although in Brazil, having sexual relations with someone under the age of 14 years [44] is considered “a crime”, almost one-eighth of adolescents already had their first sexual relation. Furthermore, very young adolescents are just beginning adolescence, adapting to the physical changes caused by puberty and often lack knowledge about reproductive health. Therefore, there is a need to assure the rights of VYA so that they can have a full and safe development towards adulthood. Prevention and detection of violence and abuse are certainly a strategy to achieve such purpose. Thus, our findings reinforce the importance of sex education programs that include gender inequalities, rights, sexual violence and communication skills, especially in low- and mid-income countries. These strategies delay sexual initiation and increase the use of contraceptive method in the first sexual intercourse. Timely interventions in sexual education that integrate family and schools may be the key to avoiding adverse events of the sexual initiation in adolescents, specifically, in girls, whose adverse events, as unintended pregnancy, can increase the risk of diverse health outcomes towards the mother and the newborn.
